# Proliferation and differentiation potential of chondrocytes from osteoarthritic patients

**DOI:** 10.1186/ar1709

**Published:** 2005-03-03

**Authors:** Tommi Tallheden, Catherine Bengtsson, Camilla Brantsing, Eva Sjögren-Jansson, Lars Carlsson, Lars Peterson, Mats Brittberg, Anders Lindahl

**Affiliations:** 1 Department of Clinical Chemistry and Transfusion Medicine, Sahlgrenska University Hospital, Gothenburg, Sweden; 2 Department Orthopaedics, Sahlgrenska University Hospital, Gothenburg, Sweden

## Abstract

Autologous chondrocyte transplantation (ACT) has been shown, in long-term follow-up studies, to be a promising treatment for the repair of isolated cartilage lesions. The method is based on an implantation of *in vitro *expanded chondrocytes originating from a small cartilage biopsy harvested from a non-weight-bearing area within the joint. In patients with osteoarthritis (OA), there is a need for the resurfacing of large areas, which could potentially be made by using a scaffold in combination with culture-expanded cells. As a first step towards a cell-based therapy for OA, we therefore investigated the expansion and redifferentiation potential *in vitro *of chondrocytes isolated from patients undergoing total knee replacement. The results demonstrate that OA chondrocytes have a good proliferation potential and are able to redifferentiate in a three-dimensional pellet model. During the redifferentiation, the OA cells expressed increasing amounts of DNA and proteoglycans, and at day 14 the cells from all donors contained type II collagen-rich matrix. The accumulation of proteoglycans was in comparable amounts to those from ACT donors, whereas total collagen was significantly lower in all of the redifferentiated OA chondrocytes. When the OA chondrocytes were loaded into a scaffold based on hyaluronic acid, they bound to the scaffold and produced cartilage-specific matrix proteins. Thus, autologous chondrocytes are a potential source for the biological treatment of OA patients but the limited collagen synthesis of the OA chondrocytes needs to be further explained.

## Introduction

Adult articular cartilage consists of a delicate system of cells and matrix proteins, which have the function of creating a viscoelastic tissue with high biomechanical stability and low friction. Even though the cartilage is exposed to continuous mechanical wear, there is surprisingly low turnover in cells and extracellular matrix [1], which could be a reason for the inability of adult articular cartilage to respond to injuries and subsequently repair lesions. This low potential of self-repair has led to the development of several techniques such as mosaic plastic, microfracture, periosteal transplantation and autologous chondrocyte transplantation (ACT), all seeking to create a functional and painless repair of articular cartilage defects.

In ACT, culture-expanded chondrocytes are transplanted under a cover of periosteum [2]; the method was initially aimed at the treatment of small isolated lesions. However, 10 years later, the indication has been expanded to include lesions up to 20 cm^2 ^in size. This first generation of cell-based treatment has been followed by a second or third generation, consisting of culture-expanded cells loaded on a membrane or into a biodegradable scaffold before implantation [3,4]. One major advantage in using scaffolds as cell carriers is that the cells can be positioned in the lesion, thereby ensuring that the cells become evenly distributed in the defect. Subsequently, the degradation time of the scaffold needs to be controlled. This can be made by different combinations of poly-L-lactic acid and poly-(lactic-co-glycollic acid) [5] or by the esterification of hyaluronic acid [6,7]. The scaffold made of hyaluronic acid has additionally been shown to degrade into chondrogenically active components [8].

Another major advantage of using a scaffold for delivery of the cells is the potential for treating larger defects. This is especially interesting for young (under 60 years old) and active patients with developed osteoarthritis (OA), who at present lack an appropriate treatment alternative. The aetiology of OA has been suggested to contain a phenotypic alteration of the chondrocytes [9] and disturbance in the proteoglycan metabolism due to systematic, mechanical or unknown reasons. Chondrocytes isolated from OA cartilage have been shown to be more metabolically active than cells isolated from non-OA regions in the same joint [10], whereas chondrocytes isolated from less severe grades of OA cartilage synthesize normal matrix components [11].

When chondrocytes are isolated from their three-dimensional (3D) environment in the articular cartilage and expanded in monolayer cultures, the cells dedifferentiate and gradually lose their specific phenotype [12,13]. We have shown previously that dedifferentiated cells from ACT patients have the ability to differentiate into several mesenchymal phenotypes [14] and that during redifferentiation towards the chondrogenic phenotype the cells express genes known to be involved in the embryonic formation of cartilage [15].

We therefore proposed, as a first step towards cell-based treatments for OA, that culture-expanded cells from patients diagnosed for OA have the capacity to proliferate and produce matrix proteins in the same quantity as ACT chondrocytes when placed in a differentiation model.

## Materials and methods

### Cartilage harvest

Cartilage biopsies were harvested with a curved chisel from macroscopically affected and unaffected surplus cartilage from seven patients with OA (age 64 to 83 years), with OA grades 3 to 5 on the Ahlbäck scale [16], undergoing total knee replacement. The affected side was considered to be the femoral condyle on the concave side of the knee deformity; that is, the medial condyle in varus deformity and the lateral in valgus knees. In all patients the hip–knee–ankle angle was determined from standing whole-leg radiographs (an angle of more than 180° indicates a valgus knee deformity). The harvested biopsies were transported to the cell culture laboratory in sterile saline solution (0.9% NaCl; Fresenius Kabi, Uppsala, Sweden) supplemented with gentamicin sulphate (50 mg/l; Gibco, Paisley, Renfrewshire, UK) and amphotericin B (250 μg/ml; Gibco). Part of the cartilage biopsy was processed for histology, blinded and scored by two independent experienced researchers in accordance with a modified (biopsies without subchondral bone) Mankin scale [17], with a maximum score of 13. The rest of the biopsy was used for cell culture as described below. The donation of surplus cartilage was approved by the ethical committee at the Medical Faculty at Gothenburg University.

### Cell culture

The chondrocytes were isolated from the surrounding matrix by mechanical mincing of the tissue with scalpel followed by enzymatic treatment overnight with collagenase (0.8 mg/ml; Worthington Biochemical Corp, Lakewood, NJ, USA) in Ham's F-12 medium (Invitrogen, Lidingö, Sweden), at 37°C in 7% CO_2_/93% air. The isolated cells were seeded at 10^4 ^cells/cm^2 ^in culture flasks (Costar; Corning Incorporated, Corning, NY, USA) in DMEM/F12 medium (Invitrogen) supplemented with L-ascorbic acid (0.025 mg/ml; Apotekets produktionsenhet, Umeå, Sweden), gentamicin sulphate (50 mg/l; Gibco), amphotericin B (250 μg/ml) and L-glutamine (2 mM; Gibco) with the addition of 10% human serum [18]. In brief, the human serum was collected in transfusion bags (dry pack; JMS, Singapore) from healthy blood donors. The serum was left to coagulate overnight at 4 to 8°C, centrifuged, sterile filtered, divided into aliquots and frozen until use. The first medium change was made on day 6 and thereafter twice a week. When the cells reached 80% confluence, they were subcultured and frozen. Thawed cells were subcultured into new flasks (Costar) at a density of 4 × 10^3 ^cells/cm^2^.

### Three-dimensional pellet culture

After passage 1, the cells were cultured in a 3D pellet culture system as described previously [15,19]. On days 7 and 14, the pellets were fixed in Histofix™ (Histolab Products AB, Göteborg, Sweden), dehydrated and embedded in paraffin. Sections 5 μm thick were cut and placed on microscope slides (Superfrost Plus; Menzel-Gläser, Braunschweig, Germany), deparaffinized and stained with Alcian blue/van Gieson or immunohistochemically with anti-type I collagen and anti-type II collagen antibodies.

### Immunohistochemistry of pellets

Deparaffinized sections were digested with hyaluronidase, 8,000 units/ml (Sigma, St Louis, MO) in 0.1 M PBS for 60 min at 37°C and blocked with 3% BSA (Sigma) in PBS for 5 min. The primary antibodies (anti-type I and II collagen; ICN Biomedicals, Aurora, OH, USA), diluted 1:150 in PBS containing 3% BSA, were incubated with the sections for 1 hour at room temperature (20–22°C). The secondary antibody, peroxidase-conjugated goat anti-mouse (1:150; Jackson Immunoresearch Laboratories, West Grove, PA, USA) were applied to the sections for 1 hour at room temperature. A substrate kit (Vector VIP; Vector Laboratories, Burlingame, CA, USA) was used for visualization and the results were analysed with a Nikon Optiphot2-pol microscope (Nikon Instruments Inc, Melville, NY, USA). Goat cartilage and bone explants were used as a positive control; for a negative control the primary antibodies were omitted.

### Biochemical analysis of pellets

On days 7 and 14, pellets were digested in papain (Sigma) solution (0.3 mg/ml in 20 mM sodium phosphate buffer, pH 7.4, containing 1 mM EDTA and 2 mM dithiothreitol) for 60 min at 60°C. The digested pellets were then mechanically dissolved by vortex-mixing and further analysed for DNA, glycosaminoglycan and hydroxyproline content as described previously [15]. All biochemical analyses was performed on triplicate pellets.

### Cells in scaffold

Culture-expanded cells (passage 2), 10^6^/cm^2 ^or 5.0 × 10^6^/cm^2^, were seeded on human serum precoated Hyaff-11 scaffolds (thickness 2 mm; Fidia Advanced Biopolymers, Abano Terme, Italy) in 100 μl in Ham's F12 medium (Invitrogen) supplemented with 20% human serum. After incubation overnight at 37°C in 7% CO_2_/93% air, the scaffolds were cultured in serum-free medium [15] in non-adherent dishes (Falcon four-well IVF; Becton Dickinson, Le Pont De Claix, France) for 14 days. After fixation, the scaffolds were embedded in paraffin, sectioned (10 μm thickness), stained with Alcian blue/van Gieson and analysed immunohistochemically for type II collagen as described above.

### Isolation of total RNA

Total RNA was isolated from cells cultured in a monolayer (passage 1) and from day 7 pellets with the use of an RNeasy mini kit (Qiagen, Hilden, Germany) in accordance with the manufacturer's description. Before RNA isolation, the pellets were collected in a 1.5 ml micro-tube (Sahrstedt, Nümbrecht, Germany) containing RLT buffer (Qiagen) and disrupted by sonication. To remove cell debris and cartilage matrix proteins a QIAshredder column (Qiagen) was used. Contaminating genomic DNA was removed from the isolated RNA by using a DNA-free kit (Ambion, Huntingdon, UK) and total RNA content and purity were determined spectrophotometrically at 260 and 280 nm. In general, *A*_260_/*A*_280 _ratios of about 2 were considered to indicate acceptable purity of the samples [20].

### Real-time PCR

Expression patterns of four cartilage genes were analysed by real-time PCR with an ABI PRISM 7000 (Applied Biosystems, Foster City, CA, USA) sequence detector and software system. TaqMan MGB probes (FAM dye-labelled) and primers for type I collagen (Hs00164004_m1) and type X collagen (Hs00166657_m1) were ordered from Applied Biosystems assays-on-demand (20× assay mixes). The gene-specific primers and probes for type II collagen 5'-TGG TGT CAA AGG TCA CAG AGG TTAT-3', antisense 5'-GGA ACC ACT CTC ACC CTT CACA-3', probe 5'-TCC CTT AGC ACC GTC CAG GCC TG-3', were designed by using Primer Express Software version 2.0 (Applied Biosystems). All genes were designed to amplify fragments of 70 to 150 base pairs; as endogenous control, 18S rRNA labelled with VIC/TAMRA was used (Applied Biosystems).

Reverse transcription *in vitro *was performed with 500 ng of total RNA with the use of random hexamer primers and TaqMan Reverse Transcription reagents (Applied Biosystems). Real-time PCR was performed with 5 μl of diluted (1:10) cDNA corresponding to 10 ng of RNA, 15 μl of TaqMan Universal PCR master mixture (Applied Biosystems), 1× assay-on-demand mixes of primers and TaqMan MGB probes. All samples were analysed in triplicate and PCR was performed in optical 96-well microtitre plates (Applied Biosystems). After an initial denaturation step at 95°C for 10 min, the cDNA products were amplified with 40 PCR cycles consisting of a denaturation step at 95°C for 15 s and an extension step at 60°C for 1 min.

To analyse the real-time PCR data, a standard curve method was used. The data were analysed with ABI Prism 7000 SDS software (Applied Biosystems). For each sample, the *Ct*_sample _values were determined as the cycle number at which all samples were in the exponential phase of amplification. By using the formulas below, a value (*Y*) was obtained as a measure of the gene expression correlated to the standard curve for that particular gene: *X *= (*Ct*_sample _- Intercept value)/Slope value; *X*^10 ^= *Y*. The *Y *value for each cDNA sample and target sequence was divided by the *Y *value from the housekeeping gene (18S) for that particular sample to derive a Δ*Ct *value (PE-ABI; Sequence Detector User Bulletin 2).

### Statistical analysis

Biochemical differences between donors and chondrocytes isolated from affected and unaffected were analysed with a two-sided Student's *t*-test (two-sample equal variance). *P *< 0.05 was considered significant. All analyses were performed with cell samples from at least three separate donors unless otherwise indicated; as a comparison, surplus cells from three or four donors undergoing ACT were used [15].

## Results

**Figure 1:**
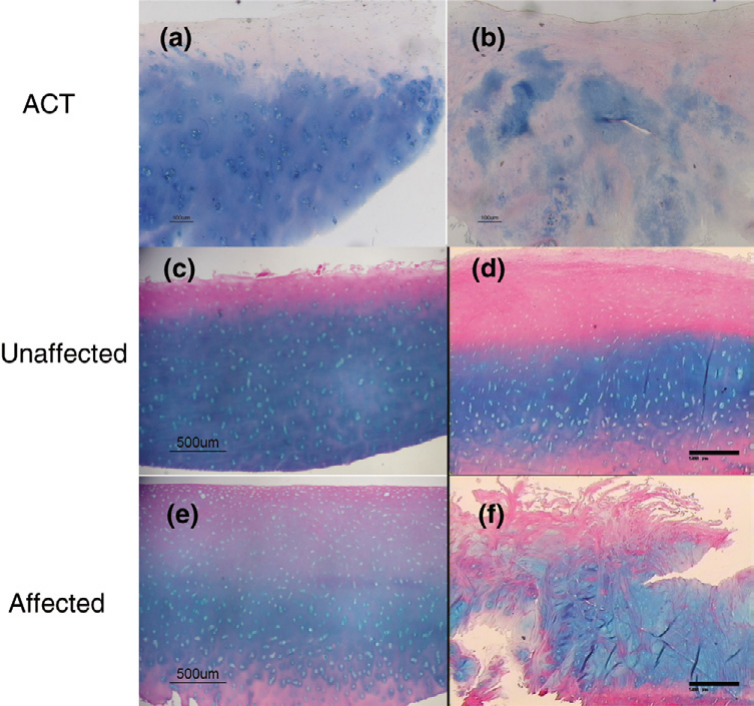
Histology of biopsies. The figure shows sections stained with Alcian blue/van Gieson. The biopsies originate from two representative autologous chondrocyte transplantation patients (ACT) **(a, b) **and from the unaffected and affected side from two patients with osteoarthritis (OA) undergoing total knee replacement **(c–f)**. **(c, e) **Biopsy from one OA donor (female, aged 81 years) with a Mankin score of 1.5 on both sides. **(d, f) **Biopsy from another OA donor (male, aged 74 years) with Mankin scores of 5 (d) and 11 (f).

After histological preparation, four of the seven isolated biopsies were evaluated on the Mankin scale for severity of OA [17]. The score in these samples varied from 1.5 to 11, and in two of the patients a significant difference was found between the affected pathological and unaffected non-pathological side of the joint (Fig. 1). After mechanical and enzymatic isolation of the chondrocytes from the biopsies, no difference could be observed in the average number of cells per milligram of cartilage between the affected and unaffected sides (Table 1). These numbers did not differ from the average number of cells isolated from ACT patients [21].

In the primary cultures of the isolated chondrocytes from the unaffected and affected sides, floating matrix fragments were initially found in the affected cultures. These fragments did not seem to affect the proliferation ability and disappeared after the first change of medium. The cells from the unaffected and affected sides expanded with, on average, 0.21 and 0.22 cell doublings per day, respectively. After one passage (4.3 cell doublings) and 3 weeks of culture, 10^6 ^primary cells isolated from a 400 mg biopsy were expanded into 20 million cells.

**Figure 2:**
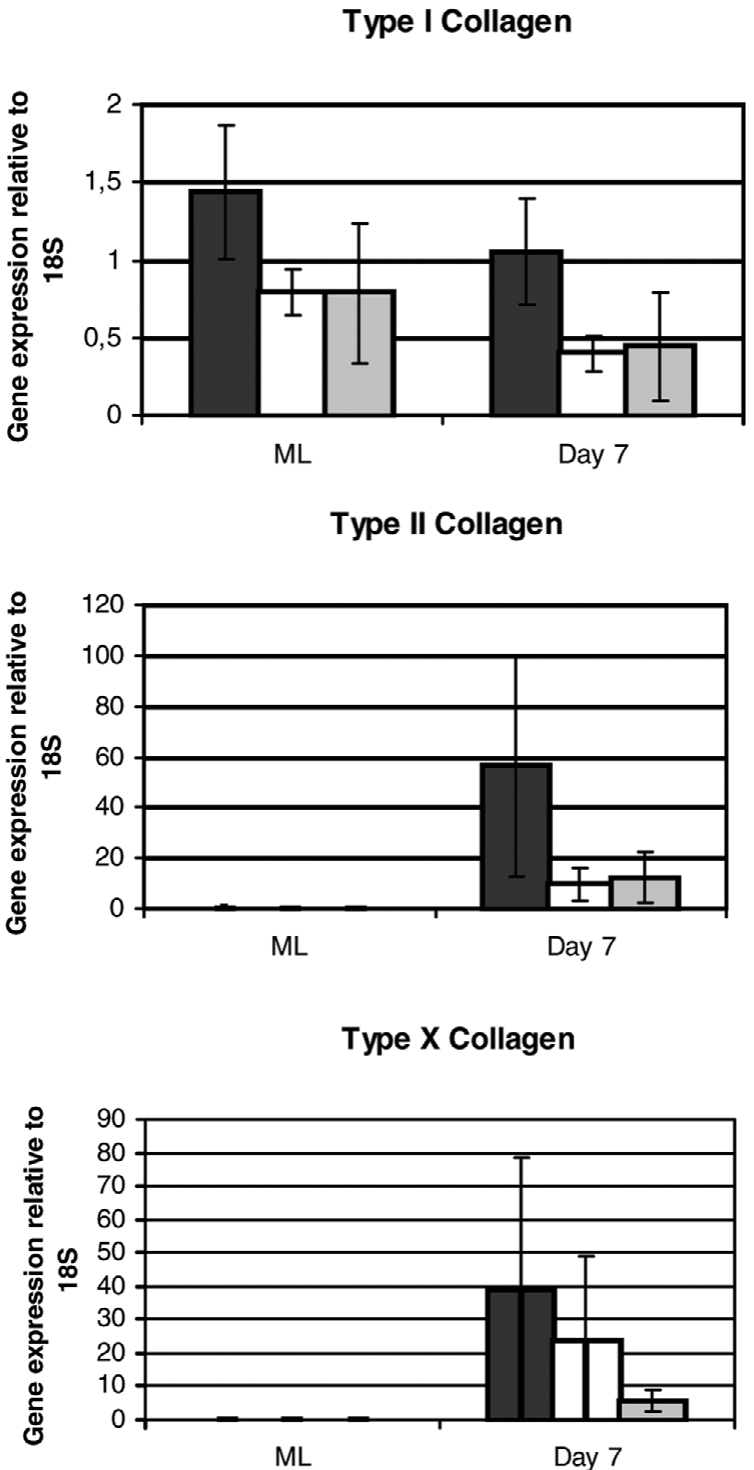
Gene expression of cells cultured in pellets. The graphs show the quantitative gene expression of typical cartilage gene expression markers (types I, II and X collagen) in the monolayer (ML) and in day 7 pellets. Results are means ± SD for separate donors (*n *= 4) from samples from autologous chondrocyte transplantation patients (black bars) and from affected (grey bars) and unaffected (white bars) areas.

When the expanded cells were cultured in serum-free medium in a redifferentiation model they formed spherical pellets overnight. During this shift from two-dimensional culture to 3D culture, the cells expressed increasing amounts of type II and type X collagen, whereas the expression of type I collagen was unchanged or slightly decreased (Fig. 2). No difference in expression of these typical cartilage genes was observed between affected, unaffected and ACT donors.

**Figure 3:**
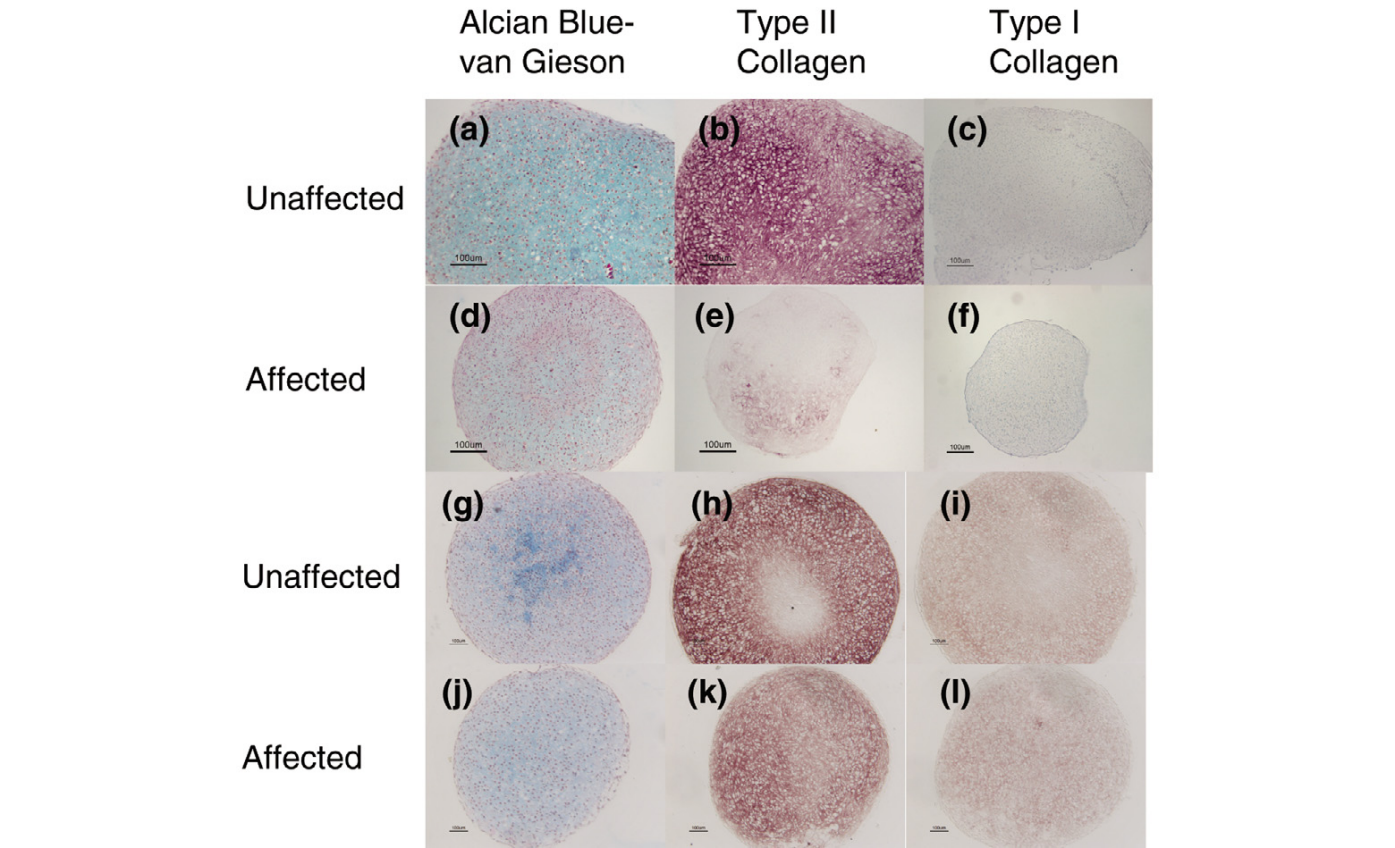
Histology of pellets. The figure shows stained sections of pellets from the unaffected and affected side from same OA donors (**(a–f) **female, age 81 years and **(g–l) **male, age 74 years) as shown in Fig. 1. **(a, d, g, f) **Alcian blue–van Gieson staining indicating the accumulation of proteoglycans. **(b, e, h, k) **Immunohistochemical staining with anti-type II collagen antibody. **(c, f, i, l) **Immunohistochemical staining with anti-type I collagen antibody. Positive staining is indicated by a red colour in the extracellular matrix.

The shift from a proliferative to a matrix-synthesizing state was also demonstrated by an increase in the size of pellets from day 1 to day 14. The histological sections of these pellets showed flattened cells on the surface and round cells in the centre (Fig. 3). Spindle-shaped cells were found in the central part of the pellet in some donors, and the frequency of spindle-shaped cells was greater in samples isolated from biopsies with high Mankin scores. In the pellets, sulphated proteoglycans were detected by Alcian blue/van Gieson staining at both days 7 and 14 in all donors (Fig. 3). Metachromatic staining was normally found throughout the whole pellets, but slightly weaker staining was found in the day 7 pellet from the sample with the highest Mankin score (data not shown).

**Figure 4:**
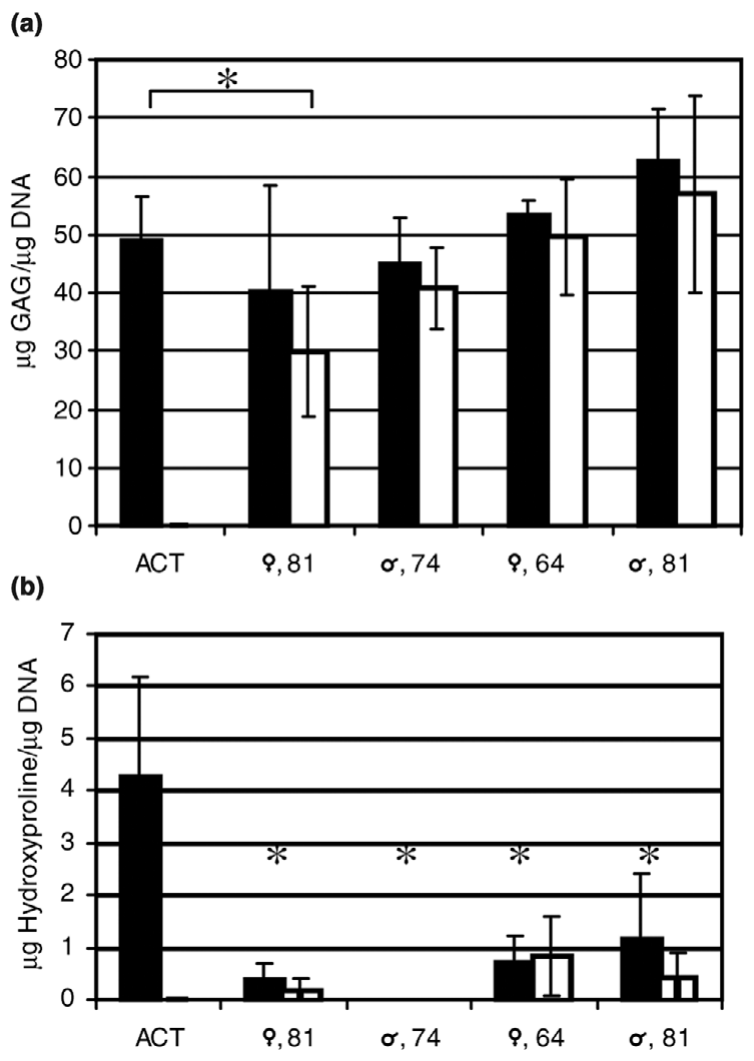
Biochemical analysis of pellets. Results are shown of the measurement of cartilage matrix protein accumulation in day 14 pellets normalized to cell number (DNA) in cells from the unaffected (filled bars) and affected (open bars) sides of four consecutive donors; the sex and age (in years) of the donors are indicated. Glycosaminoglycan (GAG) **(a) **and hydroxyproline (as a measure of total collagen content) **(b) **are shown as amounts per microgram of DNA. Results are means ± SD for three identical pellets. The amounts are compared with the mean value for four sequential autologous chondrocyte transplantation patients (ACT). An asterisk indicates a significant difference (*P *< 0.05) between ACT and osteoarthritis chondrocytes.

The increase in pellet size during the culture period was accompanied by a significant increase in DNA amounts in all samples, except from one donor with Mankin score 11 on the affected side, between days 7 and 14 (data not shown). During the same period there was an increase in proteoglycan accumulation in each cell in 63% of all samples. At day 14 no difference could be observed in the amount of proteoglycans per cell in pellets from the unaffected and affected sides (*P *> 0.05). In a comparison between OA chondrocytes and those from patients undergoing ACT only one sample, from the affected side of a female aged 81 years, had a significantly lower content of proteoglycans (*P *< 0.05; Fig. 4a).

The ability of the culture-expanded cells to form collagens in the pellet model was analysed biochemically and by immunoreactivity to type I and type II collagen (Fig. 3). The total amounts of collagen per cell were significantly lower in all OA samples than in those from ACT patients (Fig. 4b). By immunohistochemistry, type II collagen was detected in all donors at day 14, on both affected and unaffected sides, without any correlation with Mankin score. In the immunohistochemical analysis for type I collagen, both samples from one donor (a male aged 74 years) with Mankin scores of 6 (unaffected) and 11 (affected), stained positive at days 7 and 14. The other samples were only weakly positive at day 14.

**Figure 5:**
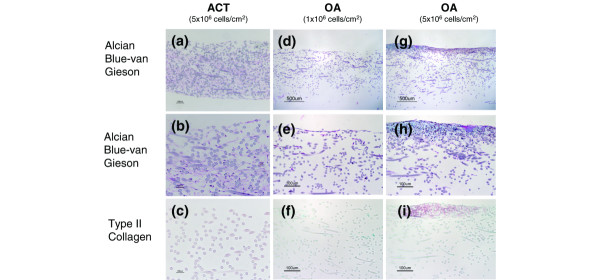
Histology of cell-seeded scaffolds. The figure shows scaffolds seeded with chondrocytes from one representative autologous chondrocyte transplantation (ACT) donor **(a–c) **and one osteoarthritis (OA) donor (unaffected) at two different cell seeding densities, 10^6 ^cells/cm^2 ^**(d–f) **and 5 × 10^6 ^cells/cm^2 ^**(g–i)**. Accumulation of proteoglycans is shown with Alcian blue/van Gieson stain and the presence of type II collagen is indicated by red staining within the scaffold. Panels (b), (e) and (h) are higher magnifications of portions of (a), (d) and (g), respectively.

To test the potential of using Hyaff-11 as a scaffold for the delivery of chondrocytes, the scaffold was seeded from the top with two different concentrations of cell suspensions of OA samples and samples from ACT patients. After the use of this technique, the chondrocytes could be detected throughout the whole thickness of the scaffold, but higher concentrations of cells were observed on the side of the scaffold from which the cells had been seeded (Fig. 5). This cell distribution was more obvious in the scaffolds seeded with OA chondrocytes than in those seeded with ACT chondrocytes.

Attached to the hyaluronic acid, the chondrocytes redifferentiated within the scaffold, as seen by the secretion of proteoglycans and the synthesis of type II collagen (Fig. 5). The expression of cartilage proteins was more obvious on the surface of the scaffolds seeded with the high cell density than those seeded with the low cell density, as shown by the increased intensity in staining with Alcian blue/van Gieson and in staining for type II collagen (Fig. 5).

## Discussion

Chondrocytes isolated from OA cartilage are able to proliferate in a monolayer and redifferentiate in 3D models, demonstrating properties similar to those of non-OA chondrocytes used for ACT. This indicates that culture-expanded autologous chondrocytes from OA patients could potentially be used for resurfacing articular cartilage.

In this paper we studied the potential of chondrocytes isolated from patients with developed OA. During the initial monolayer culture, chondrocytes are extracted from their normal 3D environment and exposed to an artificial environment consisting of a plastic surface, culture medium and serum. The plastic provides a substrate for the growth of the anchorage-dependent cells and the culture medium stabilizes pH and osmolarity and supplements the cells with trace compounds and energy sources (pyruvate and glucose). The added serum contains high levels of growth factors released by cells and platelets during the coagulation process of whole blood and has the ability to stimulate cell proliferation [18]. In this artificial environment enriched in growth factors the chondrocytes proliferate, dedifferentiate and lose their phenotype. The ability of these dedifferentiated cells to redifferentiate into the chondrogenic phenotype has been proven to be affected by the growth factors used during expansion [22] and the number of cell divisions [23].

In ACT treatments, 10^6 ^cells/cm^2 ^are implanted into the defects under a covering of periosteum or type I/III collagen membrane [24]. If this treatment were to be used for OA patients, most probably both the femoral condyle and the tibial plateau would need restoration. This would mean that surfaces about at least 25 cm^2 ^in size should be treated. In the present study we were able to obtain, from a 400 mg cartilage biopsy taken from OA patients, 20 million cells within 3 weeks of culture. This means that without exceeding the number of cell divisions, which could possibly hamper the redifferentiation potential [25], it would be necessary to harvest about 500 mg of cartilage, which correlates to a circular biopsy 7.2 mm in diameter on the basis of calculations of normal hyaline cartilage [26]. The data in this study indicate that the biopsy could be harvested either from a non-weight-bearing area or from the actual affected area during a cleanout pre-arthroscopic procedure. However, 10^6 ^cells/cm^2 ^greatly exceeds the cell density in adult cartilage, and the number of cells actually needed for a successful scaffold-assisted cartilage repair has not been defined.

It has previously been demonstrated in several studies that cells isolated from OA cartilage have limited proliferation capacity [27] and malfunctioning proteoglycan synthesis [10,11]. It was therefore a great surprise to us that we observed a proliferation rate similar to that in samples from patients treated with ACT and no difference in the proteoglycan secretion in chondrocytes isolated from affected and unaffected areas. All samples had the further ability to produce type II collagen in the pellet model. Possible explanations for this are that during the proliferation phase the cells are exposed to an environment and to growth factors, which 'revitalizes' the cells [10], or simply that there is a positive selection of potent cells during the monolayer culture.

Although the chondrocytes from OA patients analysed in this study produced a cartilage-specific matrix, the ability of the chondrocytes to redifferentiate seemed be different from that of chondrocytes isolated from ACT donors [15]. Whereas ACT chondrocytes, once placed in the 3D serum-free pellet model, stopped their DNA synthesis and started to differentiate, the OA chondrocytes continued to proliferate up to day 14. The proliferation was accompanied with significantly less collagen production in all OA chondrocytes than in ACT chondrocytes (Fig. 4b). A shift from a differentiated phenotype to a proliferative state has further been suggested as an explanation for the development of OA [9,28] and could possibly be reflected in the inability to redifferentiate seen in the pellet model.

Another important issue in cell-based cartilage repair, especially for large defects, is the positioning of cells in large defects. This can be done by delivering the cells to the patient within a vehicle or a scaffold. Within the scaffold, which is preferably biodegradable and has a controlled degradation time, the cells are able to attach and to start producing cartilage matrix. In our study we observed that, within the hyaluronic acid scaffold after 2 weeks of culture in serum-free medium, the OA chondrocytes formed cartilage matrix proteins. This result concurs with previous studies with human epiphyseal chondrocytes and chick embryonic sternal chondrocytes, in which an increased expression of cartilage typical genes was observed in cultures with Hyaff-11 (scaffold based on hyaluronic acid) [29].

The redifferentiation of the dedifferentiated cells was typically more obvious in the scaffolds seeded with the high density of cells (25 × 10^6 ^cells/cm^3^), indicating that the cell density is important for the restoration of the chondrogenic phenotype. The cell density and redifferentiation could also be important for matrix production, because in the scaffolds seeded with a low cell density (5 × 10^6 ^cells/cm^3^) we observed a threefold to fivefold lower secretion of proteoglycans compared with the pellet cultures (data not shown). Similar observations have been presented by Puelacher and colleagues [30], who showed that at least 20 × 10^6 ^cells/cm^3 ^were needed for good matrix formation within the scaffold.

Further, it is of great importance that the scaffold, when implanted into the joint, has the ability to integrate with the surrounding cartilage and with the subchondral bone. Integration with the subchondral bone could possibly be increased by the induction of subchondral bleeding, for example by microfracture. However, the importance of an uninjured subchondral bone plate for the integrity of the articular cartilage and the ability to withstand mineralization has not been clarified.

The integration could also be altered by the grade of differentiation of the scaffold, as demonstrated in a study made by Obradovic and colleagues [31]. They showed that the integration of tissue-engineered cartilage to articular cartilage explants was better with immature (redifferentiated for 5 days) than mature (redifferentiated for 5 weeks) cartilaginous explants. The positive immunostaining of type II collagen seen in our scaffold seeded with the higher density of cells could indicate that the cells had redifferentiated too far and that the implant would therefore be less integrative. In contrast, in the treatment of large injuries, the scaffold needs to be able to withstand mechanical load and shear forces from the time of implantation. These forces can possibly be lowered by alignment of the mechanical axis (tibia osteotomy) to reduce the weight bearing of the implant, but the scaffold will in any case be subjected to mechanical stress and will have to be able to withstand this. A possible way of strengthening the scaffold without redifferentiation would be to distribute the chondrocytes more uniformly in the scaffold by improving the seeding method. Both spinner flask and perfusion culture techniques have been shown to be superior to static cultures [32].

Another reason that the implant has to withstand mechanical stress is that systematic redifferentiation signalling, as part of the disease condition, could be impaired within the OA joint. Redifferentiation and proteoglycan synthesis could instead be stimulated by dynamic mechanical compression of the implant. The mechanical load could possibly be gradually increased to adapt to the differentiation state of the implant through specifically developed physiotherapy programmes, which will therefore probably have an important role in the development of biological implants for OA.

## Conclusion

We demonstrate in this paper that OA chondrocytes have the ability to proliferate, redifferentiate and secrete cartilage-specific matrix proteins. We also show that OA chondrocytes have an inability to shift definitely from a proliferative to a differentiating state. How to change the cells from a proliferative to a collagen-secreting phenotype needs to be explored further, especially when considering the importance of collagen in maintaining the cartilage structure.

We further showed that the OA chondrocytes are able to bind to a scaffold, but further studies will be needed to establish how far the cartilage in this scaffold should be differentiated to be able to integrate with the surrounding cartilage and subchondral bone and to withstand the mechanical forces applied within the joint.

The results in this paper give hopes for finding a cell-based autologous biological treatment for young active patients with OA, but we have to remember that there is no normal cartilage in OA and more research must be done before such a treatment can be put into clinical practice.

## Abbreviations

3D = three-dimensional; ACT = autologous chondrocyte transplantation; BSA = bovine serum albumin; DMEM = Dulbecco's modified Eagle's medium; PCR = polymerase chain reaction; PBS = phosphate-buffered saline; OA = osteoarthritis.

## Competing interests

The author(s) declare that they have no competing interests.

## Authors' contributions

TT conceived of the study, coordinated the experiments, performed immunohistochemical staining, performed the statistical analysis and drafted the manuscript. C Bengtsson performed the cell culture and RNA preparations. C Brantsing performed immunohistochemical stainings and the quantitative PCR analysis. ESJ participated in the design of the study and gave clinical cell culturing input. LC isolated the biopsies and gave clinical feedback. LP and MB provided critical clinical input to the study design and to the manuscript. AL conceived of the study and gave critical comments on the manuscript. All authors read and approved the final manuscript.

## Figures and Tables

**Table 1:** Clinical diagnosis and histological scores of the seven donors

No.	Cartilage	Age (years)	Sex	Ahlbäck	Varus	Valgus	HKA angle	Diagnosis	Mankin	Cells/mg
1	Affected	73	Male	III	Yes		175°	Prim OA	n/a	n/a
	Unaffected								n/a	n/a
2	Affected	74	Male	IV	Yes		174°	Prim OA	n/a	2,912
	Unaffected								n/a	2,422
3	Affected	83	Female	V		Yes	184°	Prim OA	n/a	2,147
	Unaffected								n/a	2,917
4	Affected	81	Female	II		Yes	182°	Sec OA	4.5	2,054
	Unaffected								5	1,923
5	Affected	74	Male	III	Yes		176°	Prim OA	11*	1,310
	Unaffected								5.5	1,938
6	Affected	64	Female	IV	Yes		167°	Prim OA	1.5	3,465
	Unaffected								1.5	3,091
7	Affected	81	Female	IV	Yes		173°	Prim OA	9*	2,649
	Unaffected								6.5	2,410

An asterisk indicates a significant difference (*P *< 0.05) between unaffected and affected biopsies. HKA angle, hip–knee–ankle angle; n/a, not analysed; OA, osteoarthritis; Prim, primary; Sec, secondary.
